# The appropriateness of antimicrobial use in the outpatient clinics of three hospitals in the Netherlands

**DOI:** 10.1186/s13756-020-0689-x

**Published:** 2020-02-22

**Authors:** Annemieke K. van den Broek, Reinier M. van Hest, Kamilla D. Lettinga, Afra Jimmink, Fanny N. Lauw, Caroline E. Visser, Jan M. Prins

**Affiliations:** 1 Department of Internal Medicine, Division of Infectious Diseases, Amsterdam UMC, University of Amsterdam, Meibergdreef 9, 1105 AZ Amsterdam, The Netherlands; 2 Department of Hospital Pharmacy, Division of Clinical Pharmacology, Amsterdam UMC, University of Amsterdam, Meibergdreef 9, 1105 AZ Amsterdam, The Netherlands; 3 Department of Internal medicine, Onze lieve vrouwe gasthuis, location West, Jan Tooropstraat 164, 1061 AE Amsterdam, The Netherlands; 4 Department of Hospital Pharmacy, Onze lieve vrouwe gasthuis, location West, Jan Tooropstraat 164, 1061 AE Amsterdam, The Netherlands; 5 Department of Internal medicine, MC Slotervaart, Louwesweg 6, 1066 EC Amsterdam, The Netherlands; 6 Department of Internal medicine, MC Jan van Goyen, Jan van Goyenkade 1, 1075 HN Amsterdam, The Netherlands; 7 Department of Medical Microbiology, Amsterdam UMC, University of Amsterdam, Meibergdreef 9, 1105 AZ Amsterdam, The Netherlands

**Keywords:** Antimicrobials, Antibiotic stewardship, Inappropriate prescribing, Hospital outpatient clinics

## Abstract

**Objectives:**

Antimicrobial Stewardship Programs commonly have an in-hospital focus. Little is known about the quality of antimicrobial use in hospital outpatient clinics. We investigated the extent and appropriateness of antimicrobial prescriptions in the outpatient clinics of three hospitals.

**Methods:**

From June 2018 to January 2019, we performed ten point prevalence surveys in outpatient clinics of one university hospital and two large teaching hospitals. All prophylactic and therapeutic prescriptions were retrieved from the electronic medical records. Appropriateness was defined as being in accordance with guidelines. Furthermore, we investigated the extent to which the dose was adjusted to renal function and documentation of an antibiotic plan in the case notes.

**Results:**

We retrieved 720 prescriptions for antimicrobial drugs, of which 173 prescriptions (24%) were prophylactic. A guideline was present for 95% of prescriptions, of which the guideline non-adherence rate was 25.6% (*n* = 42/164) for prophylaxis and 43.1% (*n* = 224/520) for therapy. Of all inappropriate prescriptions (*n* = 266), inappropriate prescriptions for skin and soft tissue infections (*n* = 60/226) and amoxicillin-clavulanic acid (*n* = 67/266) made up the largest proportion. In only 13 of 138 patients with impaired or unknown renal function the dosage regimen was adjusted. Amoxicillin-clavulanic acid was the drug for which most often renal function was not taken into account. In 94.6% of prescriptions the antibiotic plan was documented.

**Conclusions:**

In hospital outpatient clinics, a substantial part of therapeutics were inappropriately prescribed. Amoxicillin-clavulanic acid was the most inappropriately prescribed drug, due to non-adherence to the guidelines and because dose adjustment to renal function was often not considered.

## Background

Antimicrobial resistance leads to increased morbidity, mortality and healthcare costs worldwide [1]. In order to contain antimicrobial resistance, Antibiotic Stewardship Programs (ASP) have been developed to measure and improve the appropriateness of antimicrobial use [2]. A common way to measure the appropriateness of antimicrobial use is by evaluating whether antimicrobials are prescribed according to local guidelines and if not available, to national or international guidelines [3].

ASPs are commonly focused on in-hospital therapeutic and perioperative prophylactic antimicrobial use [4]. However, up to 90% of antimicrobial use occurs in the outpatient setting, of which, next to family practice, internal medicine and paediatrics are the largest contributors [5, 6]. Available studies evaluating outpatient antibiotic use addressed therapeutic antimicrobial use in the ambulatory setting in general, of which 30–50% was inappropriately prescribed [7–9]. However, due to the variety of clinical practice locations that are considered ambulatory care settings, there is little in-depth information on these settings individually, and in particular the appropriateness of antimicrobial prescribing practices in hospital-based outpatient clinics has received little attention [4]. Analysis of antibiotic utilization across the spectrum of inpatient and ambulatory care would be useful to direct antibiotic stewardship efforts [10]. Also, mainly antibiotics have been investigated. Antifungal and antiviral drug resistance is emerging and should therefore not be overlooked when measuring the appropriateness of antimicrobial use [11–14].

The aim of this study was to quantify the extent and appropriateness of therapeutic and prophylactic antimicrobial prescribing at the outpatient departments of a tertiary and two secondary hospitals, during ten point prevalence surveys (PPS) [15] in each hospital. Appropriateness was measured using established and validated quality indicators, of which the prescription being in accordance with the guideline was our main parameter [3].

## Materials and methods

### Study design and setting

The study was performed in the outpatient departments of three hospitals in The Netherlands, covering the period June 2018 to January 2019. The participating hospitals were Amsterdam UMC, location Academic Medical Center (AMC), a 1000-bed university-affiliated tertiary care hospital with > 300,000 outpatient clinic visits per year, the Onze Lieve Vrouwe Gasthuis Hospital, location West (OLVG W), a 225-bed secondary care hospital, treating 200,000 outpatients a year; and the MC Slotervaart (SLZ) a 150-bed secondary care hospital with 90,000 outpatient clinic visits per year. An ASP was present in all hospitals, including an Antibiotic stewardship team (AST) consisting of an infectious diseases specialist, hospital pharmacist and medical microbiologist. Approval from the Ethics committee was not required for this study because we used data for quality optimization purposes. The procedures were in accordance with the General Data Protection Regulation [16].

### Data collection and procedures

We performed in each hospital ten point-prevalence surveys (PPS) [15] on consecutive workdays to generate a representative sample size. Prior to the PPS, we developed an algorithm for the electronic medical records (EMR) of the hospitals that generated all prescriptions of the Anatomical Therapeutic Chemical (ATC) groups A02B, A07A, J01, J02, J04, J05, P01 and P02, per day and per outpatient clinic. The AMC and OLVG W utilize EPIC as EMR and SLZ utilizes Chipsoft. The EMR reports were verified on completeness by comparing the electronically generated data with data retrieved by manually checking all patient files of the outpatient departments, during 3 days for AMC and SLZ, and during 1 day for the OLVG W, because the EMR report of that hospital had already been used and validated for other purposes. If the reports showed to be incomplete, the algorithms were adjusted, after which a re-run followed until the manually collected results and the electronically collected results corresponded for at least 90%.

During the PPS, we collected the antimicrobial prescriptions of all outpatients aged 16 year or above. We excluded the outpatient clinics of paediatrics and neonatology and prescriptions of peri-operative prophylaxis, antiretroviral therapy and hydrochloroquine, since the latter is only used in the Netherlands as an anti-rheumatic drug.

The data collected were the number of antimicrobial prescriptions per outpatient department, the type of antimicrobial agent (ATC), dosage and duration of therapy and the route of administration. For each prescription we collected data from the patients’ EMR about the diagnosis and indication for prescribing, which we categorized into therapeutic indications and prophylactic indications (medical prophylaxis versus post-surgical/intervention prophylaxis, i.e. prophylaxis lasting > 24 h after the intervention). If the indication of the prescription was not clearly documented in the patient files, we contacted the treating physician. Next, we checked the presence of local guidelines (antimicrobial guidelines derived from national guidelines, with adjustments made according to resistance patterns in the hospital) and if not available, national (by the Dutch Working Party on Antibiotic Policy, www.swab.nl, or by professional societies) or international guidelines. If the prescription differed from the recommended first choice therapy in the guideline, we contacted the treating physician for a clarification. In case the clarification made clear that the deviation from the first choice therapy was justified, the prescription was labelled as appropriate. An example is a deviation from the first choice therapy because of intolerance for the first choice agent which was not documented in the EMR. We checked whether the antibiotic plan was documented in the case notes. Finally, we retrieved the renal function of each subject so that it could be checked whether dose adjustment because of impaired renal function was indicated.

### Study endpoints

The primary endpoints were the amount of antimicrobial prescriptions in hospital outpatient clinics, both therapeutic and prophylactic, and the appropriateness of these prescriptions, expressed as the percentage of antimicrobial prescriptions that were prescribed according to the available guidelines. The secondary endpoint was the percentage of antimicrobial prescriptions with documentation of the antibiotic plan in the case notes and the percentage of antimicrobial prescriptions with correct adjustment of the dosage regimen to renal function.

### Data analysis

The proportion of prophylactic prescriptions was expressed as percentage of the total number of antimicrobial prescriptions. The proportion of guideline (non)-adherent prescriptions was expressed as percentage of the total number of antimicrobial prescriptions for which a guideline was present. We evaluated whether antimicrobial therapy or prophylaxis was indicated according to the guideline, whether the right antimicrobial agent was chosen and whether the right dose and duration of therapy was prescribed. Prescriptions that differed from the recommended first choice agent in the guideline because of former culture results or known intolerances/allergies were considered appropriate. The reason of non-adherence (treatment/prophylaxis not indicated, inappropriate agent, inappropriate dose/duration) was presented as percentage of the total number of antimicrobial prescriptions that did not adhere to the guideline. Prescriptions were reviewed by one investigator, and in case of uncertainties discussed with the other investigators (antimicrobial stewardship team members of the three hospitals).

The proportion of prescriptions with a documented antimicrobial plan was expressed as percentage of the total number of prescriptions. The proportion of prescriptions with an appropriately adjusted dosage regimen in case of renal impairment was presented as percentage of the total number of prescriptions for which dose adjustment was recommended in case of renal impairment, according to the national SWAB guidelines (Dutch Working Party on Antibiotic Policy) [17]. The renal function had to be obtained within 6 months prior to the PPS and was otherwise reported as renal impairment unknown. These latter prescriptions were added to the denominator. Because the prevalence of patients with an eGFR< 10 is expected to be low, we excluded from this analysis antimicrobial drugs that are only recommended to be adjusted in patients with an eGFR< 10, to avoid overestimation of the non-adherence rate for dose adjustment in renal impairment. Since this was an exploratory study, only descriptive statistics were presented, for which IBM SPSS statistics version 25 was used.

## Results

### Characteristics of antimicrobial prescriptions

The total number of outpatient antimicrobial prescriptions retrieved during the ten point prevalence surveys of the three hospitals combined was 720, all prescribed by medical specialist and medical specialists in training. Antibiotics (ATC-code J01) were the most commonly prescribed drugs and accounted for 569 (79%) prescriptions. Table 1 presents the characteristics of the antimicrobial prescriptions per hospital. The proportion of prophylaxis versus therapy was similar for the three hospitals. Therapeutic prescriptions accounted for 547 antimicrobial prescriptions (76%) and prophylaxis for 173 (24%). The main indication for antimicrobial therapy was skin and soft tissue infections (*n* = 144, 26.3%). The main indication for prophylaxis was medical prophylaxis (*n* = 134, 77.5%). Limited variation was seen between the hospitals in this respect (Table 1).

**Table 1:** Characteristics of antimicrobial prescriptions

	Hospital			
	AMC	OLVG W	SLZ	Total
Number of prescriptions	364	199	157	720
Antibiotics (%)	276 (75.8)	159 (79.9)	134 (85.4)	569 (79)
Antimycotics (%)	15 (4.1)	12 (6.0)	7 (4.5)	34 (4.7)
Antimycobacterials (%)	9 (2.5)	3 (1.5)	3 (1.9)	15 (2.1)
Antivirals (%)	38 (10.4)	12 (6)	4 (2.5)	54 (7.5)
Antiprotozoals (%)	20 (5.5)	5 (2.5)	2 (1.3)	27 (3.8)
Antihelmintics (%)	5 (1.4)	2 (1)	–	7 (1)
Other^a^ (%)	1 (0.3)	6 (3)	7 (4.5)	14 (1.9)
Indication for therapy (% of total prescriptions)	266 (73.1)	157 (78.9)	124 (79)	547 (76)
Skin and soft tissue (%)	65 (24.4)	50 (31.8)	29 (23.4)	144 (26.3)
Urogenital tract (%)	41 (15.4)	29 (18.5)	38 (30.6)	108 (19.7)
Respiratory tract (%)	41 (15.4)	38 (24.2)	19 (15.3)	98 (17.9)
Gastro-intestinal tract (%)	37 (13.9)	10 (6.4)	13 (10.5)	60 (11)
Ear-nose-throat (%)	26 (9.8)	9 (5.7)	10 (8.1)	45 (8.2)
Oral-maxillofacial (%)	7 (2.6)	10 (6.4)	15 (12.1)	32 (5.9)
Ophthalmology (%)	16 (6)	6 (3.8)	–	22 (4)
Other^b^ (%)	33 (12.4)	5 (3.2)	–	38 (6.9)
Indication for prophylaxis (% of total prescriptions)	98 (26.9)	42 (21.1)	33 (21)	173 (24)
Medical prophylaxis (%)	85 (86.7)	34 (81)	15 (45.5)	134 (77.5)
Surgical/intervention prophylaxis (%)	13 (13.3)	8 (19)	18 (54.5)	39 (22.5)

*Abbreviations: AMC* Amsterdam UMC, location Academic Medical Center, *OLVG W* Onze Lieve Vrouwe Gasthuis Hospital, location West, *SLZ* MC Slotervaart

^a^Other: *H. pylori* eradication, ^b^Other: < 10 prescriptions

**Fig. 1:**
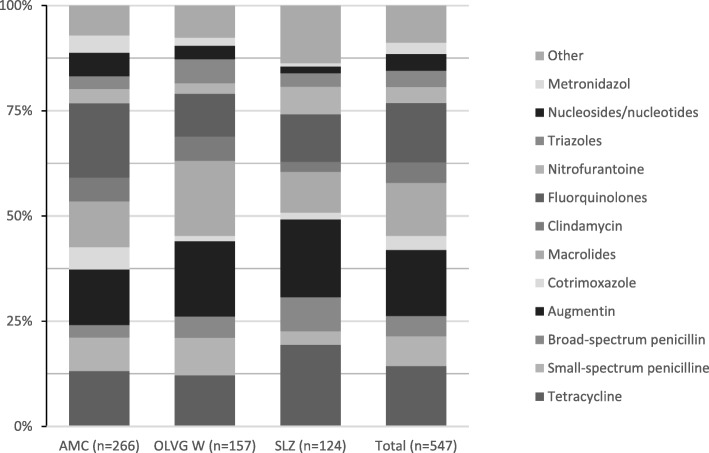
Antimicrobial prescriptions - Therapy

**Fig. 2:**
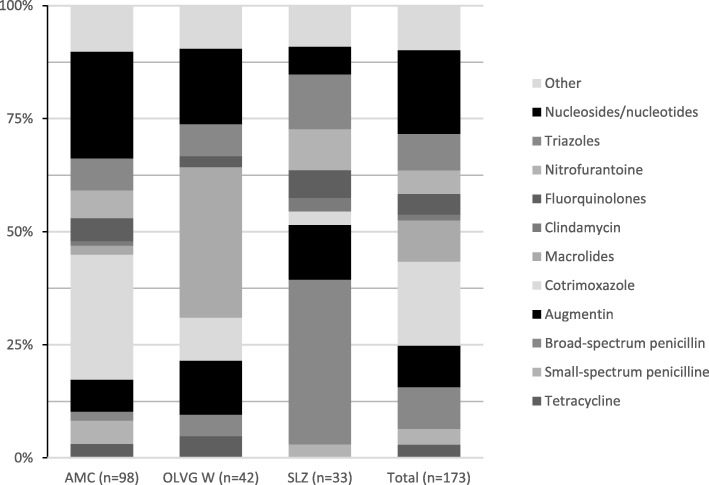
Antimicrobial prescriptions - Prophylaxis

Figures 1 and 2 show the distribution of the prescribed antimicrobial agents per indication (therapy and prophylaxis) and per hospital. For therapeutics, the distribution of prescribed agents was comparable for the three hospitals. For prophylaxis, cotrimoxazole and nucleosides and nucleotides (excluding HIV reverse transcriptase inhibitors) were the most commonly prescribed (both *n* = 32, 18.5%). However, the distribution of prescribed prophylactic antimicrobials varied between the hospitals. Cotrimoxazole represented the largest group of prophylactic antibiotics in the AMC (tertiary care university hospital). AMC has a large HIV, haematology and nephrology department, where patients receive kidney- and stem cell transplantations and other extensive haematology immunosuppressive treatment. In these patients cotrimoxazole is often used. Of the 27 cotrimoxazole prescriptions, 26 were for such patients. Macrolides were the most used prophylactic antibiotics in the OLVG W. A possible explanation could be that, unlike the other hospitals, the PPS in this hospital were performed during the winter, when macrolides are used as prophylaxis for COPD patients [18, 19]. Broad spectrum penicillins represented the largest group of prophylactic antibiotics in the SLZ, which corresponds with the extent of post-surgical intervention prescriptions (Table 1).

### Appropriateness of antimicrobial prescriptions - guideline adherence

Table 2 shows an overview of the guideline adherence, separated for therapy and prophylaxis. A guideline was present for most prescriptions (*n* = 684(95%), of which *n* = 488 local guidelines), and this did not differ between prophylaxis and therapy. Altogether, 266 (38.9%) prescriptions did not adhere to the guideline. Of the prescribed therapeutics, 224 (43.1%) were inappropriate, mainly because the choice of agent or dose/duration were not in compliance with the guideline. Of the prescribed prophylaxis, 42 (25.6%) were inappropriate, mainly because there was no indication for prophylaxis. Guideline adherence varied between the hospitals. The presence or absence of local guidelines, with national/international guidelines coming in place when local guidelines are absent, was not statistically significant associated with the adherence rate (χ2-test, *p*-value: 0.21).

**Table 2:** Guideline adherence

	Therapy				Prophylaxis				Total
	AMC	OLVG W	SLZ	Total	AMC	OLVG W	SLZ	Total	
Number of prescriptions	266	157	124	547	98	42	33	173	720
Guideline available (%)	249 (93.6)	149 (94.9)	122 (98.4)	520 (95.1)	96 (98)	36 (85.5)	32 (97)	164 (94.8)	684 (95)
Guideline non-adherence (%)	95 (38.2)	84 (56.4)	45 (36.9)	224 (43.1)	16 (16.7)	7 (19.4)	19 (59.4)	42 (25.6)	266 (38.9)
No indication (%)	17 (17.9)	13 (15.5)	10 (22.2)	40 (17.9)	15 (93.8)	6 (85.7)	18 (94.7)	39 (92.9)	79 (29.7)
Inappropriate agent (%)	31 (32.6)	37 (44)	24 (53.3)	92 (41.1)	–	–	–	–	92 (34.5)
Inappropriate dose/duration (%)	47 (49.5)	34 (40.5)	11 (24.4)	92 (41.1)	1 (6.3)	1 (14.3)	1 (5.3)	3 (7.1)	95 (35.7)

Guideline adherence per indication and per antimicrobial agent are presented in Tables 3 and 4 respectively. Overall, prescriptions for skin and soft tissue infections (*n* = 60, 22.6%) and amoxicillin-clavulanic acid (*n* = 67, 25.2%) made up the largest proportion of guideline non-compliant prescriptions. For skin and soft tissue infections, this was most often because of an inappropriate dose or duration of therapy (*n* = 43, 71.7%) and for amoxicillin-clavulanic acid because the choice of the agent was not recommended by the guideline (*n* = 38, 56.7%). Variation was seen between the hospitals. For instance, in the SLZ prescriptions for post-surgical prophylaxis were more often inappropriate (*n* = 18, 28.1%) and in the OLVG W, macrolides were more often inappropriately prescribed (*n* = 21, 23.1%).

**Table 3:** Guideline non-adherence per indication

	Hospital			
	AMC	OLVG W	SLZ	Total
Respiratory tract (%)	15 (13.5)	27 (29.7)	6 (9.4)	48 (18)
Gastro-intestinal tract (%)	10 (9)	2 (2.2)	3 (4.7)	15 (5.6)
Urogenital tract (%)	8 (7.2)	14 (15.4)	14 (21.9)	36 (13.5)
Skin and soft tissue (%)	26 (23.4)	28 (30.8)	6 (9.4)	60 (22.6)
Ear-nose-throat (%)	25 (22.5)	7 (7.7)	7 (10.9)	39 (14.7)
Oral-maxillofacial (%)	2 (1.8)	3 (3.3)	9 (14.1)	14 (5.3)
Ophthalmology (%)	5 (4.5)	2 (2.2)	–	7 (2.6)
Other (%)	4 (3.6)	1 (1.1)	–	5 (1.9)
Medical prophylaxis (%)	5 (4.5)	2 (2.2)	1 (1.6)	8 (3)
Surgical/intervention prophylaxis (%)	11 (9.9)	5 (5.5)	18 (28.1)	34 (12.8)
Total	111	91	64	266

**Table 4:** Guideline non-adherence per antimicrobial agent

	Hospital			
	AMC	OLVG W	SLZ	Total
Tetracycline (%)	6 (5.4)	7 (7.7)	2 (3.1)	15 (5.6)
Small-spectrum penicillin (%)	18 (16.2)	11 (12.1)	3 (4.7)	32 (12)
Broad-spectrum penicillin (%)	4 (3.6)	6 (6.6)	16 (25)	26 (9.8)
Amoxicillin-clavulanic acid (%)	24 (21.6)	19 (20.9)	24 (37.7)	67 (25.2)
Cotrimozaxole (%)	5 (4.5)	2 (2.2)	1 (1.6)	8 (3)
Macrolides (%)	11 (9.9)	21 (23.1)	6 (9.4)	38 (14.3)
Clindamycin (%)	7 (16.3)	7 (7.7)	1 (1.6)	15 (5.6)
Fluoroquinolones (%)	18 (16.2)	10 (11)	4 (6.3)	32 (12)
Nitrofurantoin (%)	3 (2.7)	1 (1.1)	2 (3.1)	6 (2.3)
Triazoles (%)	4 (3.6)	4 (4.4)	1 (1.6)	9 (3.4)
Nucleosides/nucleotides (%)	8 (7.2)	2 (2.2)	1 (1.6)	11 (4.1)
Metronidazole (%)	1 (0.9)	–	–	1 (0.4)
Other (%)	2 (1.8)	1 (1.1)	3 (4.7)	6 (2.3)
Total (%)	111	91	64	266

### Appropriateness of antimicrobial prescriptions – documented plan and dosage adjustment

A documented plan was available in 94.6% (range between hospitals: 89.8–97.5%) of the prescriptions.

There were 138 antimicrobial prescriptions for which dosage adjustment was recommended because of renal impairment (Table 5). Of these, only 13 (9.6%) were adjusted. The antimicrobial agent in which most frequently renal function was not taken into account was amoxicillin-clavulanic acid: of all amoxicillin-clavulanic acid prescriptions (*n* = 102), 62.7% was incorrectly not adjusted, which accounted for 50% of the not-adjusted prescriptions in patients with impaired or unknown renal function.

**Table 5:** Prescriptions adapted to renal function

	Hospital			
	AMC	OLVG W	SLZ	Total
Nr. of prescriptions to be adjusted to renal function	63	41	34	138
Nr. adjusted	12	1	–	13
Nr. not adjusted	51	40	34	125
Antimicrobial agents not adjusted (% of total^a^)				
Amoxicillin-clavulanic acid (*n* = 102)	22	22	20	64 (62.7)
Cotrimoxazole (*n* = 50)	4	1	0	5 (10)
Macrolides (*n* = 85)	3	4	1	8 (9.4)
Fluoroquinolones (*n* = 85)	12	9	5	26 (30.6)
Nitrofurantoin (*n* = 30)	4	2	4	10 (33.3)
Triazoles (*n* = 85)	1	1	1	3 (8.6)
Nucleosides/nucleotides (*n* = 54)	5	1	2	8 (9.3)
Other (*n* = 65)	–	–	1	1 (1.5)

^a^*n* = total number of prescriptions for that agent, regardless of renal function

## Discussion

We investigated the prescription rate and appropriateness of prophylactic and therapeutic antimicrobials in the outpatient clinics of one tertiary care university hospital and two secondary care hospitals. In the outpatient clinics a quarter of the antimicrobials were prescribed for prophylaxis. We identified several targets for quality improvement projects. Although guidelines were present for most prescriptions (95%), these were not followed in a substantial proportion of cases (38.9%). Of these, mainly therapeutic antibiotics were inappropriately prescribed, which contributed for 84.2% to the total inappropriate prescriptions. Amoxicillin-clavulanic acid was the most frequent inappropriately prescribed antimicrobial agent, due to non-adherence to the guideline and also because dosage adjustment in case of renal impairment was often not applied. An antimicrobial plan was present in the case notes of most prescriptions.

The overall proportion of prophylaxis prescribed in hospital outpatient clinics was similar to what is reported in the hospital wards in the recent global PPS (25.2%) [20]. To the best of our knowledge, the average proportion of prophylaxis in the ambulatory setting is unknown. Inappropriately prescribed prophylaxis made up only 15.8% of the inappropriate prescriptions. Although in the outpatient clinics the majority of the prophylaxis was indicated for medical prophylaxis, still almost a quarter of prophylaxis were prescribed for post-surgical/intervention prophylaxis. In general, prolonged use of surgical prophylaxis has not been associated with better clinical outcome, but rather with emerging antimicrobial resistance and *Clostridium difficile* infections [21, 22]. Therefore, prophylaxis that is continued after 24 h is in general considered inappropriate. This explains our findings: prophylaxis that was not in compliance to the available guidelines was primarily due to unnecessarily prescribed post-surgical/intervention prophylaxis.

Of all therapeutic prescriptions 43.1% did not adhere to the guideline, mainly due to an inappropriate choice of antimicrobial agent or dose/duration of therapy, which is almost twice as much as was reported for hospital wards (22.6%) [20]. Previous studies addressing the appropriateness of antibiotic prescriptions in the ambulatory care setting described a non- adherence rate similar to ours. However, in these studies it was unclear whether it also included prophylaxis [7, 9]. Our results showed that prescriptions for skin and soft tissue infections (SSTI) were the most frequently inappropriate, while previous studies in the ambulatory care setting mainly showed inappropriate prescriptions for respiratory tract infections [7, 8, 23, 24]. Antibiotic use for respiratory tract infections is seasonal driven [25]. In two of the three hospitals the PPS were performed during the summer [25]. Also, it is conceivable that consultations for respiratory tract infections are more common in general practice than in hospital outpatient clinics. Finally, antibiotic use for respiratory tract infections has received extensive attention from ASPs, which might have led to less inappropriate prescriptions [7, 8, 23, 24]. In previous studies it was already shown that antimicrobial treatment of uncomplicated SSTI had a low guideline adherence rate, 11–20.2%, due to an inappropriate length of treatment and due to an inappropriate choice of broad spectrum antibiotic agents [9, 26, 27]. Altogether, these findings suggest that there is considerable room for quality improvement for SSTI prescriptions and emphasize the need of information on antibiotic use per clinical care setting to direct ASP efforts [10, 24].

The main focus of ASP should be the use of amoxicillin-clavulanic acid. Amoxicillin-clavulanic acid (ACA) has become the most frequently used antimicrobial agent globally [12, 28–30]. The high use of ACA has been directly linked to an increased antimicrobial resistance, of which the resistance of *Klebsiella pneumoniae* and *Escherichia coli* to ACA has become a significant and clinically relevant problem [12, 31]. Our findings showed that ACA not only was the most frequently prescribed antimicrobial agent in hospital outpatient clinics, but also the most often inappropriately prescribed, which was also reported in previously performed PPS on hospital wards [32, 33]. In addition, we showed that when ACA was prescribed, dosage in case of renal impairment was often not adjusted, while the dosage should be adjusted in case of an estimated glomerular filtration rate below 30 ml/min. Previous reports have shown that restricting ACA use effectively reduces ACA resistance [31, 34]. In Croatia, this restriction has led to a decrease of *E.coli* resistance from 37 to 11% [34]. Altogether, we found opportunities for ASP to enhance the quality of ACA use, for patients’ safety and ACA resistance.

There are several possibilities that could explain the prescribing behaviour of antimicrobials in hospital outpatient clinics and why the non-adherence rate in the outpatient clinics was twice as high as what was observed in the hospital wards [23]. First, in hospital outpatient clinics patients have to be seen, diagnosed and treated within a short time frame and because of the time constraints clinicians might not be able to search for the guideline. Second, due to the inability of daily observing the clinical outcome of the patient, it is possible that clinicians are more cautious and prone to prescribe broad spectrum antimicrobials such as ACA, or prolonged surgical prophylaxis. Third, it is possible that clinicians are habituated to certain treatment practices which have proven to be effective, regardless of whether they are in accordance with current guidelines, and are therefore less motivated to change this habit. Further qualitative studies should be performed to elucidate the reasons of this high non-compliance rate.

### Strengths and limitations

This study has several strengths and limitations. The PPS were performed on ten different time points, in all adult hospital outpatient clinics of three different hospitals. Therefore, we were able to detect a certain pattern, rather than a local observation. However, the three hospitals were localized in the same geographic area and therefore we do not know to which extent our data is nationally or internationally representative. A strength is that we used the EMR to generate the data regarding antimicrobial use, which we validated manually in all three hospitals. Hereby, we reduced the risk of missing prescriptions. Although some pharmacies still accept handwritten prescriptions, which would be missed in our study, this is the exception rather than the rule. Additionally, we evaluated therapy and prophylaxis using several quality indicators, which enabled us to find several targets for quality improvement for ASPs. When we evaluated the prescriptions with regard to dosage adjustment to renal function, the prescriptions for patients of whom the renal function was unknown were labelled as inappropriate. By doing this, we may have overestimated the number of prescriptions in which the dose was incorrectly not adjusted according to renal function. However, the result that in 118 of 138 prescriptions the renal function was unknown shows that testing for renal function is often not considered, even though these agents require dosage adjustment when the renal function is impaired. We think it is important to raise awareness on this matter.

## Conclusion

In the hospital outpatient clinics, prophylaxis accounted for a quarter of the antimicrobial prescriptions and had in general a good guideline-adherence rate, with the exception of unnecessarily prescribed post-surgical/intervention prophylaxis, whereas a substantial part of the therapeutic prescriptions were inappropriate. Amoxicillin-clavulanic acid was the most inappropriately prescribed antimicrobial agent, regarding non-adherence to the guideline and also regarding the lack of considering renal function for dosage adjustment. Altogether, we believe that antimicrobials prescribed at the hospital outpatient clinics warrant ASP attention. The variation of the guideline adherence rate between the investigated hospitals, as well as the differences with prior studies addressing antibiotic use in ambulatory settings in general, emphasize that (hospital) outpatient antimicrobial use should be audited locally.

## Data Availability

The datasets used and/or analysed during the current study are available from the corresponding author on reasonable request.

## Abbreviations

ACA: Amoxicillin-clavulanic acid
AMC: Amsterdam UMC, location Academic Medical Center
ASP: Antibiotic stewardship programs
ATC: Anatomical Therapeutic Chemical
EMR: Electronic medical records
OLVG W: Onze Lieve Vrouwe Gasthuis Hospital, location West
PPS: Point-prevalence surveys
SLZ: MC Slotervaart
SSTI: Skin and Soft tissue infections
